# Structure-Based Drug Design and Characterization of Sulfonyl-Piperazine Benzothiazinone Inhibitors of DprE1 from Mycobacterium tuberculosis

**DOI:** 10.1128/AAC.00681-18

**Published:** 2018-09-24

**Authors:** Jérémie Piton, Anthony Vocat, Andréanne Lupien, Caroline S. Foo, Olga Riabova, Vadim Makarov, Stewart T. Cole

**Affiliations:** a Global Health Institute, Ecole Polytechnique Fédérale de Lausanne, Lausanne, Switzerland; b FRC Fundamentals of Biotechnology RAS, Moscow, Russian Federation

**Keywords:** DprE1 inhibitor, Mycobacterium tuberculosis, benzothiazinone

## Abstract

Macozinone (MCZ) is a tuberculosis (TB) drug candidate that specifically targets the essential flavoenzyme DprE1, thereby blocking synthesis of the cell wall precursor decaprenyl phosphoarabinose (DPA) and provoking lysis of Mycobacterium tuberculosis. As part of the MCZ backup program, we exploited structure-guided drug design to produce a new series of sulfone-containing derivatives, 2-sulfonylpiperazin 8-nitro 6-trifluoromethyl 1,3-benzothiazin-4-one, or sPBTZ. These compounds are less active than MCZ but have a better solubility profile, and some derivatives display enhanced stability in microsomal assays. DprE1 was efficiently inhibited by sPBTZ, and covalent adducts with the active-site cysteine residue (C387) were formed. However, despite the H-bonding potential of the sulfone group, no additional bonds were seen in the crystal structure of the sPBTZ-DprE1 complex with compound 11326127 compared to MCZ. Compound 11626091, the most advanced sPBTZ, displayed good antitubercular activity in the murine model of chronic TB but was less effective than MCZ. Nonetheless, further testing of this MCZ backup compound is warranted as part of combination treatment with other TB drugs.

## Abstract (precis)

Macozinone (MCZ) is a tuberculosis (TB) drug candidate that specifically targets the essential flavoenzyme DprE1, thereby blocking synthesis of the cell wall precursor decaprenyl phosphoarabinose (DPA) and provoking lysis of Mycobacterium tuberculosis. As part of the MCZ backup program, we exploited structure-guided drug design to produce a new series of sulfone-containing derivatives, 2-sulfonylpiperazin 8-nitro 6-trifluoromethyl 1,3-benzothiazin-4-one, or sPBTZ.

## INTRODUCTION

DprE1 is an essential flavoprotein of Mycobacterium tuberculosis involved in decaprenyl phosphoryl-β-d-arabinose (DPA) synthesis. DPA is the sole precursor of arabinose for production of both arabinogalactan and lipoarabinomannan (1), important components of the mycobacterial cell wall. DprE1, together with its counterpart DprE2, catalyzes the epimerization of decaprenyl phosphoryl-β-d-ribose (DPR) to DPA in a two-step mechanism. In the last decade, many inhibitors were discovered to target DprE1, which is now considered the Achilles' heel of M. tuberculosis due to its essentiality and especially to its localization in the periplasm (2). DprE1 inhibitors can be classified into two families according to their mode of action: some of them inhibit DprE1 irreversibly by forming a covalent adduct with cysteine 387 (C387) of DprE1, whereas others act as competitive noncovalent inhibitors (3).

The first covalent DprE1 inhibitors discovered were benzothiazinones (BTZ), exemplified by the lead compound, BTZ043, which is exceptionally potent with *in vitro* and *ex vivo* MIC values in the nanomolar range (4). A lead optimization campaign gave rise to PBTZ169, now known as macozinone (MCZ). It is currently the most potent BTZ compound against M. tuberculosis with an MIC of 0.3 nM (5), has completed preclinical development successfully and is now undergoing phase I and phase II clinical trials (https://www.newtbdrugs.org/pipeline/clinical). A common characteristic of the covalent DprE1 inhibitors is the presence of a nitro group on the molecule, which is essential for the mechanism of inhibition. Indeed, this nitro group is converted by DprE1 containing FADH_2_ into an extremely reactive nitroso group which specifically targets the cysteine residue at position 387 (C387) in the active site of DprE1, to form a covalent adduct and thereby irreversibly inhibits the enzyme (4–13). It has been demonstrated that the presence of C387 is essential for the activity of covalent DprE1 inhibitors (4, 14).

Apart from the covalent bond with C387, covalent DprE1 inhibitors are otherwise only maintained in the pocket by steric hindrance and Van Der Waals interactions, which explains why a simple substitution at C387 results in complete resistance of the enzyme to these compounds (3). As part of the MCZ backup program, this observation prompted us to revisit the structure-activity relationship (SAR) in order to obtain derivatives with other anchor points in the active site of DprE1, since such compounds might retain activity against C387 mutants should these arise.

Several noncovalent DprE1 inhibitors have also been found (15–24). Similar to the covalent DprE1 inhibitors, these noncovalent compounds sit in the substrate-binding pocket of DprE1 and act as competitive inhibitors. Interestingly, a class of noncovalent inhibitors, 2-carboxyquinoxalines, are active against BTZ-resistant M. tuberculosis strains with substitutions at C387 of DprE1 (14). Molecules from this family possess an essential 2-carboxylate moiety that forms key hydrogen bonds with the side chain of lysine 418 and the hydroxyl group of tyrosine 60 (18). Hence, it can be hypothesized that a composite molecule between 2-carboxyquinoxalines and MCZ could overcome resistance issues and increase specificity to the target.

Revisiting the SAR of MCZ further provides the opportunity to improve pharmacodynamics properties of MCZ such as aqueous solubility to increase its oral bioavailability, metabolic stability, and *in vivo* activity (25). It has been observed that the potency of BTZ derivatives is inversely proportional to their solubility (4, 5). Therefore, the solubility and bioavailability of the most active benzothiazinones are parameters for improvement. Factors controlling the aqueous solubility of organic molecules are complex and drug solubility issues are usually solved by a combination of empirical and rational drug design strategies. More than 20 crystal structures of DprE1 with or without inhibitors were reviewed recently to identify the structural determinants for activity and to guide rational drug design (3).

Based on our prior observations, the aim of this study was to design a new structure-guided series of MCZ derivatives with increased activity against either wild-type M. tuberculosis or its BTZ-resistant mutants, and with improved solubility, absorption, bioavailability, and metabolic stability of the compound *in vivo*. Therefore, a new series of MCZ derivatives, harboring a sulfonylpiperazine group, was designed (2-sulfonylpiperazin 8-nitro 6-trifluoromethyl 1,3-benzothiazin-4-one [sPBTZ]), synthesized, and characterized. This study identifies 11626091 as the best sPBTZ and demonstrates that this compound has a promising combination of antitubercular activity and ADME/T (Absorption, Distribution, Metabolism, and Excretion–Toxicity) properties.

## RESULTS

### Rationale.

When BTZ inhibitors bind to their target, DprE1, the sole bond formed is a covalent semimercaptal bond with the active-site cysteine residue, C387. We reasoned first that introducing a sulfonyl group into the MCZ scaffold might offer a second anchor by mimicking the carboxylate moiety of the 2-carboxyquinoxalines that acts as an H-bond acceptor with DprE1 and increases affinity of the inhibitor for the target. Second, sulfonyl groups are well characterized and present in many U.S. Food and Drug Administration (FDA)-approved drugs, in particular antimycobacterial agents, such as dapsone. Based on these observations, sulfonyl groups might increase both the solubility and bioavailability of the inhibitor *in vivo*. Lastly, the geometry imposed by sulfonyl groups opens new directions for investigation of the SAR.

### Synthesis of sPBTZ.

The synthesis of 17 sulfanyl-piperazino BTZ (sPBTZ) derivatives was performed in a two-step procedure from 2-(methylthio)-8-nitro-6-(trifluoromethyl)-4*H*-1,3-benzothiazin-4-one, as described previously (5). Its reaction with a 5-molar excess of free piperazine generated the corresponding piperazine derivative with a high yield. This scaffold was used in the reactions with different alkyl-, aryl-, or heteryl-sulfochlorides to form sulfanyl-piperazino BTZs. The compounds synthesized have different types of sulfonyl substitutions, thus allowing the structure-activity relationship to be studied. It is clear that compounds with alkyl substitutions have much better antitubercular activity, and aryl derivatives have much lower activity, a finding consistent with our previous data for piperazine-containing BTZ (PBTZ) derivatives (5).

### Hydrophilicity.

The octanol-water partition coefficient, logP, which is regarded as a suitable indicator of molecular hydrophobicity and bioavailability, was calculated for all derivatives to measure the effects of introducing a sulfonyl group on the PBTZ backbone. Interestingly, the addition of the sulfonyl group between the benzothiazinone and piperazine moieties has the tendency to decrease the calculated logP (clogP) coefficient and therefore hydrophobicity (Table 1). The sulfonylated derivative of MCZ, sPBTZ169 (11326127), which carries a sulfonyl group between the piperazine and cyclohexyl moieties, has a clogP of 3.28, whereas MCZ has a clogP of 4.31. This implies that the introduction of a sulfonyl group decreases hydrophobicity and may thus increase solubility in physiological conditions and subsequently could have an important impact on bioavailability.

**TABLE 1:**
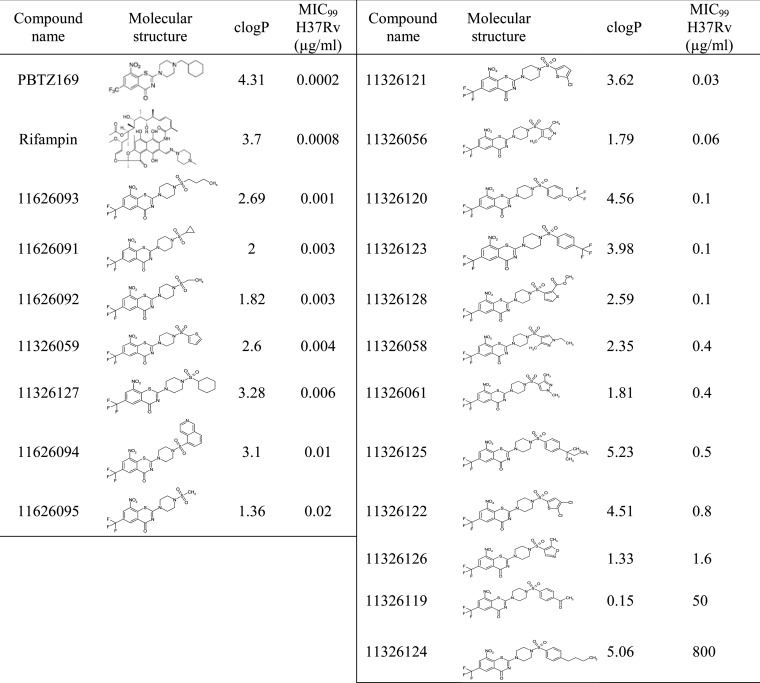
Structure-activity relationship of the different 2-sulfonylpiperazin 8-nitro 6-trifluoromethyl 1,3-benzothiazin-4-ones derivatives (sulfonyl PBTZ derivatives) in M. tuberculosis H37Rv

### Antitubercular activity.

The activity of all sPBTZ derivatives was tested *in vitro* against M. tuberculosis H37Rv and MIC_99_ values were determined (Table 1). Most sPBTZ were active *in vitro* in the submicromolar range, proving that that addition of the sulfonyl group does not abolish activity. However, none has a better activity than MCZ. 11626093, which has a butyl group, has the highest activity of MIC (1 ng/μl) corresponding to 5 times the MIC_99_ of MCZ. The introduction of a sulfone between the piperazine and the cyclohexyl negatively affected the activity of the compound, reducing activity by 30-fold, as observed with sPBTZ169 (11326127), which has an MIC_99_ of 6 nM. It was previously shown that substituting the methylcyclohexyl in MCZ with small radicals such as methyl or ethyl decreases the activity of compounds, with MICs of 250 and 60 ng/ml, respectively (5). Interestingly, when methylcyclohexyl is substituted by sulfonylmethyl (11626095) or sulfonylethyl (11626092), the activity decreases less, and the compounds are 10 times more active than the nonsulfonated derivatives (MICs of 20 and 3 ng/ml, respectively). Substitution with a butyl leads to the same activity independent of the sulfonyl group. This observation indicates that the presence of the sulfonyl group positively affects activity when the radical is small (methyl or ethyl), whereas it negatively influences activity when the substituent is long and hydrophobic.

Similarly to the other BTZ derivatives, the activities of the sulfonyl derivatives are inversely proportional to the logP (4), suggesting that activity could be related to solubility in lipids (see Fig. S1 in the supplemental material). Seven molecules were selected based on their activity/logP profile for further characterization (see Fig. S1 in the supplemental material).

### Target engagement and structural studies.

To ensure that sPBTZs specifically target DprE1, selected sulfonyl derivatives were tested against M. tuberculosis NTB1, a DprE1 mutant that carries a cysteine 387 serine (C387S) substitution and is thus resistant to BTZ. As expected, none of the sBTZs are active against NTB1, indicating that DprE1 is their primary target (Table 2). Furthermore, it is unlikely that the sulfonyl group is able to mimic the carboxylate moiety of 2-carboxyquinoxalines in stabilizing the compound in the pocket as a noncovalent inhibitor.

**TABLE 2:** *In vitro* activity of selected sPBTZ derivatives against M. tuberculosis H37Rv and its BTZ-resistant mutant (NTB1), and cytotoxicity for HepG2 cells

Compound	MIC_99_ (μg/ml)		TD_50_ (μg/ml)	Selective index
	H37Rv	NTB1		
11326059	0.004	>100	100	25,000
11326127	0.006	>100	>100	>16,666
11626091	0.003	14.7	8.10	2,700
11626092	0.003	25.1	8.90	2,967
11626093	0.001	>100	>100	>100,000
11626094	0.01	>100	100.00	10,000
11626095	0.02	75	7.80	390
PBTZ169	0.0002	>100	44.00	220,000
Rifampin	0.0008	0.0008	100.00	125,000

**FIG 1:**
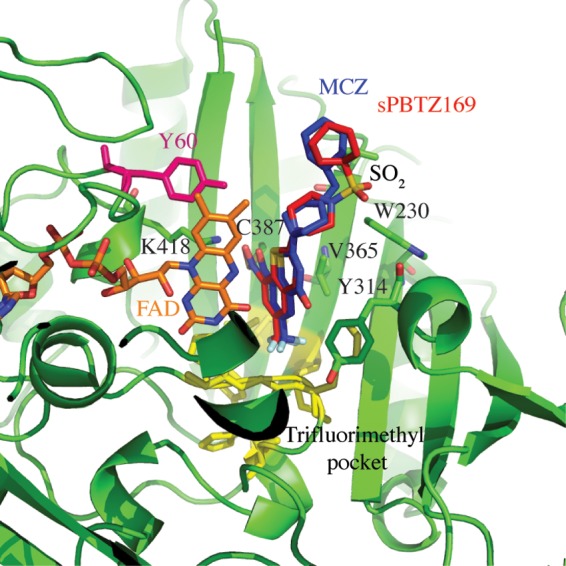
Structural comparison between the crystal structure of DprE1 in complex with sulfonyl derivative sPBTZ169 (11326127) and the structure in complex with MCZ (PDB code 4NCR) (5). DprE1 is represented in green in the illustration. sPBTZ169 (in red) sits in the trifluoromethyl hydrophobic pocket (in yellow) and binds covalently to C387. It is maintained by FAD (orange) and some lateral chains represented in sticks. Y60, a key residue in the binding of 2-carboxyquinoxalines, is represented in pink.

To investigate whether the introduction of the sulfonyl group could influence the position of the inhibitor and allow more contact within the binding pocket, a crystal structure of DprE1 in complex with sPBTZ169 was solved and compared to the crystal structure of DprE1 with MCZ. sPBTZ169 is located in the same pocket as MCZ and other BTZ derivatives. It sits in an hydrophobic pocket via the trifluoromethyl group and is covalently bound to C387 (Fig. 1). The compound is maintained by Van der Waals interaction on each side by V365, Y314, W230, and flavin adenine dinucleotide (FAD), and a hydrogen bond between K418 and the oxygen atom of the nitro group of sPBTZ169. Unfortunately, the orientation of sPBTZ does not favor the formation of a new anchor to the protein for instance interaction between the sulfonyl function and Y60. Furthermore, as MCZ, the electron density map does not account fully for the sulfonyl-cyclohexyl moiety of sPBTZ169, likely due to its higher flexibility demonstrating that sulfonyl is not stabilized in the pocket (5).

To determine whether the sulfonyl group could affect the activity at the protein level and even help to stabilize the inhibitor in the active site of a BTZ-resistant C387S DprE1 variant, the inhibition of DprE1 activity was measured *in vitro*. The 50% inhibitory concentrations (IC_50_s) were determined for the wild-type and the BTZ-resistant C387S mutant enzymes for MCZ and sPBTZ169 (see Table S2 in the supplemental material). sPBTZ169 has IC_50_s of 1.1 and 12 μM against wild-type and BTZ-resistant DprE1, respectively, whereas the corresponding IC_50_s for MCZ are 0.3 and 3.6 μM. That introducing the sulfonyl group on the MCZ scaffold leads to 4-fold less activity suggests that even if the environment of the protein is favorable for an H-bond acceptor, the presence of a hydrophobic group, such as a cyclohexyl in MCZ, is still preferable for the activity of the drug. On the other hand, the higher IC_50_ against C387S compared to the WT enzyme validates the structural studies in that the sulfonyl group does not help to stabilize the molecule in the pocket of the BTZ-resistant mutant C387S.

### ADME/T.

To assess potential cytotoxic effects of the sulfonyl group on the sPBTZ derivatives, viability of HepG2 cells was monitored after exposure to different concentrations of the compounds. The concentration for half-maximal cytotoxicity (TD_50_) was determined for each compound. Four of the seven compounds were not cytotoxic (11326059, 11326127, 11626093, and 11626094) while three of them showed mild cytotoxicity at concentrations of around 10 μg/ml (11626091, 11626092, and 11626095). Taken together, the selective index representing the ratio of the antitubercular activity of compounds (MIC_99_ against H37Rv) to cytotoxicity (TD_50_ against HepG2 cells) is more than acceptable for the chosen seven compounds (Table 2).

Since solubility issues are often encountered in drug development, which would consequently impact bioavailability, activity *in vitro*, ADMET results, and activity *in vivo*, the solubility of these derivatives was calculated by the shake flask method in equilibrium in water. Experimental solubility constants measured were then compared to theoretical solubility constants calculated using the SwissADME webserver (26). sPBTZs are predicted to have increased solubility compared to MCZ in water (Table 3) and experimental solubility constants measured in water for all sPBTZ derivatives are consistent with theoretical constants calculated by SwissADME using different algorithms (Table 3). As expected, 11626095, 11626092, and 11626091, harboring methyl, ethyl, and cyclopropyl groups, respectively, are the most soluble sPBTZ derivatives. In contrast, 11326059, 11326127, 11626093, and 11626094 that carry bigger hydrophobic groups are at least 100 times less soluble in water. Interestingly, there is a good correlation between water solubility and clogP. However, a discrepancy between the predicted solubility and experimental solubility for MCZ was observed. In fact, MCZ is 400 times more soluble in water than as calculated with different algorithms.

**TABLE 3:** Water solubility of selected sPBTZ derivatives measured by shake flask method compared to solubility calculated using SwissADME^*a*^

Compound	Solubility (μg/ml)		clogP
	Measured in water	Calculated in water	
11326059	<0.01	0.12	2.6
11326127	<0.01	0.26	3.28
11626091	8.6 ± 3.8	4.69	1.91
11626092	10.7 ± 4.6	7.2	2
11626093	0.105 ± 0.05	0.93	2.69
11626094	0.088 ± 0.04	0.18	3.1
11626095	10.6 ± 1.8	16.9	1.36
PBTZ169	31.1 ± 6.4	0.0795	4.31

a Daina et al. (26).

Another possible issue in the development of MCZ might be metabolic instability (5). In order to test whether the sulfonyl group could improve metabolic stability effected by mouse CD-1 or human liver enzymes, microsomal stability experiments were conducted. Intrinsic clearance (CL_int_) values were then calculated for each compound and compared to the CL_int_ of carbamazepine and nifedipine, used as controls for low and high intrinsic clearance, respectively. It is important to note that the compounds 11626095, 11626091, and 11626092 harboring the smallest radical chains methyl, ethyl, and cyclopropyl, respectively, have the lowest clearances in both mouse and human microsomes, indicative of their stability (Table 4). 11626093 carrying a butyl radical is metabolically stable in human but highly unstable in mouse microsomes, suggesting that it could be a good substrate for mouse but not human microsomal enzymes. Compounds 11326059 and 11326127 have medium clearances in both mouse and human microsomes, similar to MCZ and BTZ043. Of note, 11626094 is highly unstable in the presence of human microsomes. These results reveal a strong correlation between the length of the substituent after the piperazine moiety and instability in microsomes.

**TABLE 4:** Metabolic stability of sPBTZ derivatives measured in mouse and human microsomes

Compound	CL_int_ (μl/min/mg protein)		Source or reference
	Mouse microsome	Human microsome	
11326059	18.6	16.8	This study
11326127	19	21.5	This study
11626091	2.7	9.7	This study
11626092	3	1.4	This study
11626093	42	6.4	This study
11626094	24	277	This study
11626095	0.9	1.0	This study
Carbamazepine	0.6	0.4	This study
Nifedipine	105	121.1	This study
PBTZ169	36.7	28	5
BTZ043	16.5	4.20	5

### Activity in the murine model of chronic TB.

**FIG 2:**
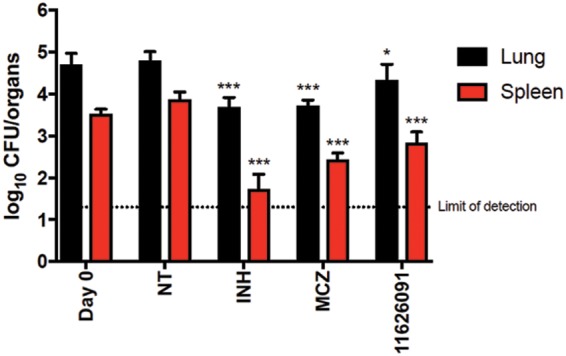
Activity of the sPBTZ derivative 11626091 (50 mg/kg) compared to *in vivo* activity of INH (10 mg/kg) or MCZ (25 mg/kg) in the mouse model of chronic TB. Black columns correspond to the bacterial burden in lungs, and red columns correspond to the bacterial burden in the spleens at day zero (Day 0) when treatment was initiated, or day 28 (NT) when treatment ended. Bars represent the means ± the standard deviations of CFU from five mice per group. NT, untreated control. Limit of detection, 20 CFU/organ. The significance of difference was calculated using a Student *t* test (*, *P* < 0.05; **, *P* < 0.005; ***, *P* < 0.0001 [compared to NT]).

Finally, the *in vivo* efficacy of 11626091 was assessed in the murine model of chronic TB following low-dose aerosol infection of BALB/cByJ mice with M. tuberculosis and treatment at 50 mg/kg (Fig. 2). Compound 11626091 was selected as it is the most promising taking into account its activity (“only” 15 times less active than MCZ), cytotoxicity, solubility, and metabolic stability properties. The *in vivo* activity of 11626091 was tested at 50 mg/kg and compared to the activity of MCZ at a standard dose of 25 mg/kg and isoniazid (INH) at a standard dose of 10 mg/kg.

Compared to the untreated control group, the bacterial burdens in the lungs and spleens of 11626091-treated mice were 0.46 (*P* < 0.05) and 1.03 (*P* < 0.0001) log_10_ CFU lower, respectively (Fig. 2), whereas the corresponding values for these organs from MCZ-treated mice were 1.03 (*P* < 0.0001) and 1.47 (*P* < 0.0001) log_10_ CFU. These results indicate that 11626091 is highly active *in vivo* in the murine model of chronic TB, although it is less able to reduce the bacterial load in the lungs than treatment with MCZ.

## DISCUSSION

Lead compounds for new anti-tuberculosis (anti-TB) drugs should possess not only potent sterilizing activity but also good pharmacokinetic profiles to facilitate their coadministration with other anti-TB and anti-HIV agents. New drugs should also be appropriate for once daily oral dosing and be relatively inexpensive to produce to ensure that all high-burden countries have access. Structure-based rational drug design supports drug development and has the potential to increase the activity and pharmacokinetic properties of lead compounds.

The new sPBTZ series was designed as part of the MCZ backup program. MCZ is a BCS (biopharmaceutical classification system) class 2 drug with a low dissolution rate but excellent absorption (5). Structural studies indicate that the environment of the binding pocket in the protein can accommodate a polar group at the cyclohexyl position of MCZ (3). A sulfonyl group was deemed to be a good candidate because it is well characterized and present in many soluble and metabolically stable FDA-approved drugs, including drugs used to treat mycobacterial infections. For instance, sulfamethoxazole is a sulfonamide drug used in co-trimoxazole prophylaxis for HIV-infected patients, demonstrating that it is compatible with antiretroviral treatment (27).

It was previously shown that there is a strong correlation between logP and BTZ activity (4). We found that introduction of a sulfonyl group into the MCZ scaffold to form the sPBTZ series decreases both logP and activity *in vitro* (Table 1). However, sPBTZ still retain potency and the presence of the sulfonyl group improved aqueous solubility for those derivatives which harbor small side chains, such as methyl, ethyl, or cyclopropyl, compared to MCZ (5). Interestingly, aqueous solubility and metabolic stability measured in microsomes indicate a better profile for sPBTZ with small groups rather than those with long hydrophobic chains or MCZ. Despite being less active than MCZ our sulfonylated PBTZ with long hydrophobic chains, methyl (11626095), ethyl (11626092), or cyclopropyl (11626091) derivatives are considered good candidates in terms of their solubility and metabolic stability profiles. The efficacy of the most active derivative of the three, 11626091, was assessed *in vivo* in the murine model of chronic TB, where relatively good activity was measured in the lungs and particularly in the spleens (Fig. 2). It is also important to note that the dissolution of 11626091 was considerably easier than MCZ in methylcellulose, the solvent used for the *in vivo* studies (technical observation [data not shown]).

One of the objectives of inserting a sulfonyl group into the PBTZ backbone was to mitigate potential BTZ resistance by increasing the number of hydrogen bonds with DprE1. Indeed, a sulfonyl could act as an H-bond acceptor in order to anchor the protein to the H-bond donors, for example, the hydroxyl group of tyrosine 60 localized in the binding pocket. This group was identified as a key player in the stabilization of 2-carboxyquinoxalines, molecules that remain active against BTZ-resistant DprE1. Unfortunately, as was observed in the crystal structure and enzymatic inhibition assays, the sulfonyl group of sPBTZ is not implicated in the binding and stabilization of the drug in the pocket as originally hypothesized. This explains the resistance of the BTZ-resistant M. tuberculosis mutant NTB1 to sPBTZ.

To conclude, our study identifies 11626091 as an active, metabolically stable, and moderately soluble molecule that is less active than MCZ *in vitro* and *in vivo*. However, given its better solubility, compound 11626091 represents an attractive backup to MCZ that should now be tested in combination with other TB drug candidates such as bedaquiline in order to assess its potential to contribute to a new regimen.

## MATERIALS AND METHODS

### Synthesis.

The synthetic route used to produce sPBTZ and related procedures are described in the supplemental material.

### Octanol-water partition coefficient logP calculation.

The logP values were calculated using the program Hyperchem 7.5 (Hypercube, Inc.).

### Bacterial strains and culture conditions.

M. tuberculosis strain H37Rv and its BTZ-resistant mutant (C387S) NTB1 were grown at 37°C with shaking in Middlebrook 7H9 broth (Difco) supplemented with 10% albumin-dextrose-catalase (ADC) enrichment, 0.2% glycerol, and 0.05% Tween 80. The *in vitro* activities against all mycobacterial strains were measured with the resazurin reduction microplate assay (REMA) by 2-fold serial dilutions of the compounds in the working bacterial culture in 96-well plates (final volume of 100 μl). After incubation for 6 days at 37°C, the bacterial viability was determined by adding resazurin for 24 h at 37°C and measuring the fluorescence of the resorufin metabolite (excitation wavelength, 560 nm; emission wavelength, 590 nm) using a Tecan Infinite M200 microplate reader. Briefly, the noise signal obtained in the blank control wells was subtracted from the fluorescence values of test samples, and the mycobacterial viabilities in each well were proportionally calculated compared to 100% growth in control wells. Bacterial viability curves and MIC_99_ values were calculated with Prism software version 7.0, using the “Gompertz equation for MIC determination” analysis (GraphPad Software, Inc., La Jolla, CA).

### Cytotoxicity studies.

The cytotoxicity of the compounds was measured as described previously against the human hepatic cell line, HepG2 (20). Briefly, cells were incubated (4,000 cells/well) with serial dilutions of compounds (2-fold dilutions; 100 to 0.1 μg/ml) in a 96-well microplate. After incubation for 3 days at 37°C, the cell viability was determined by adding resazurin for 4 h at 37°C and measuring the fluorescence of the resorufin metabolite (excitation wavelength, 560 nm; emission wavelength, 590 nm) using a Tecan Infinite M200 microplate reader. Data were corrected for background (no-cell control) and expressed as a percentage of the value for untreated cells (cells only).

Data were fitted to obtain IC_50_s, using the “log(inhibitor) versus response – variable slope” function implemented in GraphPad Prism software version 7.0. The selective index refers to the ratio of the dose of drug that causes toxicity effects (TD_50_) to the dose that leads to the desired pharmacological effect (MIC_99_).

### Water Solubility.

Water solubility measurements were performed using the shake flask method (28). Approximately 1 ml of water was added to 1 mg of compound in an Eppendorf tube, followed by incubation for 3 days at 25°C with shaking at 800 rpm. Suspensions were centrifuged at 16,100 × *g* for 10 min, and the supernatant was filtered using 0.22-μm-pore size filters. Filtrates were injected onto a high-performance liquid chromatography (HPLC) column (Dionex), and the amount of compound was quantified using a calibration curve.

### Inhibition assays, crystallography, and structural studies of DprE1 complexed with sPBTZ.

Recombinant M. tuberculosis DprE1 was overexpressed and purified as described previously (5) to obtain highly concentrated and pure protein with bound FAD. The enzyme activities in the presence of MCZ, Ty38c, and 11326127 were measured as described previously to determine the IC_50_s for both wild-type and C131S mutant DprE1 proteins (14).

For crystallization purposes, M. tuberculosis DprE1 (approximately 40 μM) was incubated for 3 h at 30°C, with 200 μM sulfonyl-BTZ 11326127 and 200 μM FPR (farnesyl phosphoribose), in 20 mM Tris (pH 8.5), 50 mM NaCl. The protein was concentrated to approximately 15 mg/ml on an Amicon centrifugal device (30,000 MWCO; Millipore). Crystals were obtained by the hanging-drop vapor diffusion method at 18°C. Experiments were set up by mixing 1 μl of the protein sample with 1 μl of the reservoir solution containing 100 mM imidazole (pH 7.2 to 7.5) and 18 to 24% polypropyleneglycol 400. Yellow crystals grew in approximately 1 to 3 days and were transferred to a cryoprotectant (reservoir solution with 25% glycerol) prior to flash-cooling in liquid nitrogen.

X-ray data were collected at SLS, beamline PROXIMA 3. Data were integrated with the program XDS (29) and processed using PHENIX (30). The structure was solved by molecular replacement using PHENIX (31) and the structure of M. tuberculosis DprE1 (PDB code 4NCR) (5) as a template. Molecular replacement was subjected to iterative rounds of refinement and rebuilding in coot (32) and PHENIX. The coordinates and structure factors have been deposited in the Brookhaven Protein Data Bank (accession number 6G83).

### Metabolic stability *in vitro*.

The intrinsic clearance (CL_int_) of compounds was measured in both mouse and human liver microsomes. Briefly, 100 μg of mouse (CD-1) or human liver microsomes (both from Invitrogen) were mixed in 0.1 M phosphate buffer (pH 7.4) containing 0.01 μl of compound dissolved in dimethyl sulfoxide (DMSO) at 10 mg/ml in a final volume of 50 μl. In parallel, an NADPH-regenerating system (Promega) was prepared in 0.1 M phosphate buffer (pH 7.4). The solutions were preincubated at 37°C for 10 min before the intrinsic clearance assessment was initiated by mixing the two solutions (50 μl of each; final compound concentration, 1 μg/ml) at 37°C. After 0, 5, 15, 30, and 60 min, the reactions were terminated by transferring 100 μl of the reaction mixture into 100 μl of acetonitrile and placing the mixture on ice for 30 min for full protein precipitation. The samples were then centrifuged at 12,000 × *g* for 10 min, and the supernatant was injected onto an HPLC column (Dionex) to quantify the amount of parent compound remaining over time. To control that the clearance is due to the enzyme activity of the microsomes and not to unspecific microsomal protein binding, mouse (CD-1) or human liver microsomes (both from Invitrogen) were mixed in 0.1 M phosphate buffer (pH 7.4) containing 0.01 μl of compound dissolved in DMSO at 10 mg/ml, followed by incubation without the NADPH-regenerating system. After 0 and 60 min, the reactions were terminated, prepared and analyzed as previously described. Carbamazepine and nifedipine at the same concentrations were used as controls for low and high intrinsic clearances, respectively.

### Antimycobacterial activity of 11626091 against chronic TB in mice.

Female BALB/cByJ mice, aged 5 to 6 weeks, were purchased from Charles River Laboratories (France). Mice were infected with a low dose aerosol (100 to 200 CFU/lung) of logarithmic-phase M. tuberculosis H37Rv bacilli and then were allocated to experimental groups and returned to their cages. Five mice were used per time point for each regimen. Treatment was initiated 4 weeks after infection.

Macozinone (MCZ), 11626091, and INH were prepared weekly in 0.5% methylcellulose and administered at 25, 50, and 10 mg/kg, respectively, by gavage 5 days a week for 4 weeks. *In vivo* efficacy of each treatment was assessed by CFU enumeration after plating dilutions of the lung and spleen homogenates on 7H10 agar plates containing 10% oleic acid-albumin-dextrose-catalase (OADC), cycloheximide (10 μg/ml), and ampicillin (50 μg/ml). Plates were incubated for 4 weeks at 37°C before CFU were enumerated. CFU counts were log_10_ transformed before analysis as the mean log_10_ CFU ± standard deviation and compared by a Student *t* test using GraphPad Prism version 7.0 software. *P* values less than 0.05 were considered statistically significant.

Experiments were approved by the Swiss Cantonal Veterinary Authority (authorization 3082) and performed between June and August 2017.

## Supplementary Material

Supplemental file 1
