# Supplementary material for: Structure-Based Drug Design and Characterization of Sulfonyl-Piperazine Benzothiazinone Inhibitors of DprE1 from Mycobacterium tuberculosis
Source: Antimicrob Agents Chemother. 2018 Sep 24;62(10):e00681-18. doi: 10.1128/AAC.00681-18 (PMC6153800; doi:10.1128/AAC.00681-18)
Supplement: Supplemental file 1 [file zac010187478s1.pdf]

SUPPLEMENTAL MATERIALS
FOR

Structure-based drug design and characterization of sulfonyl-piperazine benzothiazinone inhibitors of DprE1 from *Mycobacterium tuberculosis*

Jérémie Piton[1], Anthony Vocat[1], Andréanne Lupien[1], Caroline Foo[1], Olga Riabova[2], Vadim Makarov[2], Stewart T. Cole[1*]

[1]Global Health Institute, Ecole Polytechnique Fédérale de Lausanne, Station 19, 1015 Lausanne, Switzerland

[2]FRC Fundamentals of Biotechnology RAS, Leninsky prospekt 33-2, Moscow, 119071, Russian Federation

Synthetic route for 2-[4-(R-sulfonyl)piperazin-1-yl]-8-nitro-6-(trifluoromethyl)-4*H*-1,3-benzothiazin-4-one derivatives

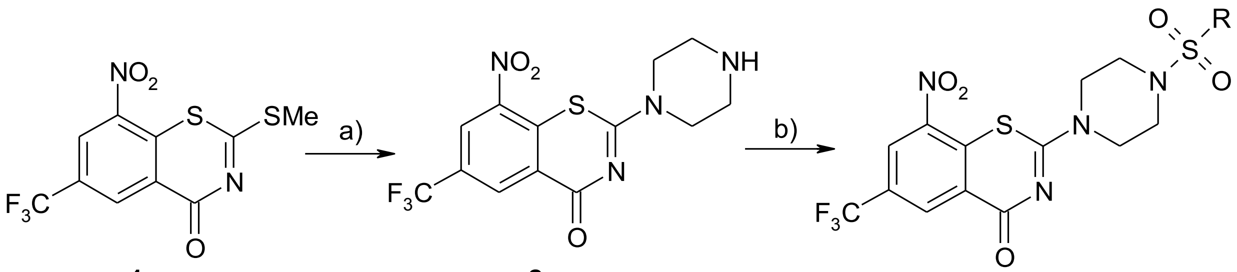

3	R	3	R
a	Me	k	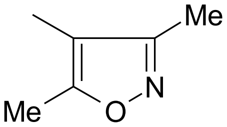
b	Et	l	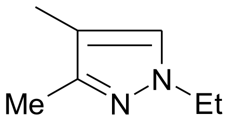
c	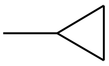	m	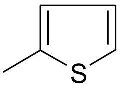
d	n-butyl	n	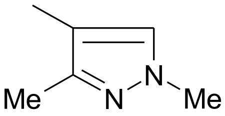
e	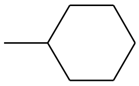	o	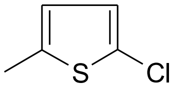
f	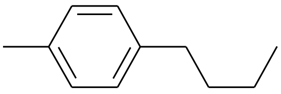	p	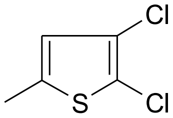
g	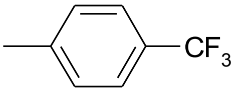	q	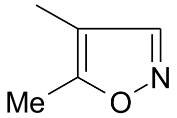
h	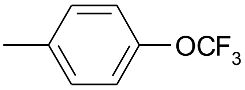	r	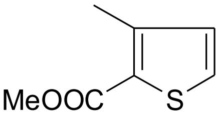
i	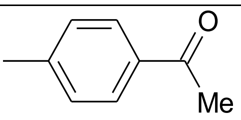	s	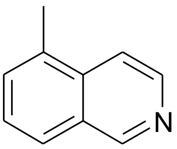
j	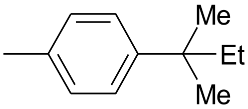		

General Procedure for the synthesis of 2-[4-(R-sulfonyl)piperazin-1-yl]-8-nitro-6-(trifluoromethyl)-4*H*-1,3-benzothiazin-4-one derivatives.

Step a). At 20 °C, finely ground solid 2-(methylthio)-8-nitro-6-(trifluoromethyl)-4*H*-1,3-benzothiazin-4-one (25 mmol) (Makarov, EMBO Mol Med, 2014) was added in one portion under intensive mixing to solution of piperazine hexahydrate (125 mmol) in 15 ml of ethanol. After 5 min reaction mixture was diluted by water, yellow solid was filtered off and recrystallised from ethyl acetate twice. The yield of 8-nitro-2-piperazin-1-yl-6-(trifluoromethyl)-4*H*-1,3-benzothiazin-4-one is 74%, mp 151-5 °C. Mass (EI), *m/z* (I_{relat}.(%)): 359.3126 $[M]^+$ (45). $C_{11}H_{13}F_3N_4O_3S$. ^1H NMR (DMSO-d_6): 3.16 (4H, br s, $N(CH_2)_2$), 3.98 (4H, br s, $N(CH_2)_2$), 8.73 (1H, s, CH), 8.87 (1H, s, CH) ppm.

Step b). Solution of 8-nitro-2-piperazin-1-yl-6-(trifluoromethyl)-4*H*-1,3-benzothiazin-4-one (10 mmol) in 5 mL of pyridine was treated by correspomding sulfonyl chloride (10 mmol) at 20 °C. After 1 hour the reaction mixture was diluted by water and acidified by concentrated hydrochloride solution in water till pH 1. Formed solid of 2-[4-(R-sulfonyl)piperazin-1-yl]-8-nitro-6-(trifluoromethyl)-4*H*-1,3-benzothiazin-4-one was filtered off and washed by water.

2-[4-(Methylsulfonyl)piperazin-1-yl]-8-nitro-6-(trifluoromethyl)-4H-1,3-benzothiazin-4-one **3a** (11626095) Yield 61%. Mp. 186-8 °C (EtOH). Mass (EI), *m/z* (I_{relat}.(%)): 437.4042 $[M]^+$ (49). $C_{14}H_{13}F_3N_4O_5S_2$. ^1H NMR (DMSO-d_6): 2.93 (3H, s, CH_3), 3.24 (4H, br s, $N(CH_2)_2$), 4.05 (4H, br s, $N(CH_2)_2$), 8.76 (1H, s, CH), 8.88 (1H, s, CH) ppm.

2-[4-(Ethylsulfonyl)piperazin-1-yl]-8-nitro-6-(trifluoromethyl)-4H-1,3-benzothiazin-4-one **3b** (11626092) Yield 74%. Mp. 204-7 °C (EtOH). Mass (EI), *m/z* (I_{relat}.(%)): 451.4208 $[M]^+$ (86). $C_{15}H_{15}F_3N_4O_5S_2$. ^1H NMR (DMSO-d_6): 1.34 (3H, t, J = 7.4 Hz, CH_3), 2.75 (2H, q, J = 7.4 Hz, CH_2), 3.26 (4H, br s, $N(CH_2)_2$), 4.06 (4H, br s, $N(CH_2)_2$), 8.77 (1H, s, CH), 8.85 (1H, s, CH) ppm.

2-[4-(Cyclopropylsulfonyl)piperazin-1-yl]-8-nitro-6-(trifluoromethyl)-4H-1,3-benzothiazin-4-one **3c** (11626091) Yield 57%. Mp. 201-4 °C (EtOH). Mass (EI), *m/z* (I_{relat}.(%)): 463.4415 $[M]^+$ (52). $C_{16}H_{15}F_3N_4O_5S_2$. ^1H NMR (DMSO-d_6): 1.48 (4H, m, CH_2-CH_2), 3.26 (4H, br s, $N(CH_2)_2$), 3.81 (1H, m, CH), 4.09 (4H, br s, $N(CH_2)_2$), 8.73 (1H, s, CH), 8.86 (1H, s, CH) ppm.

2-[4-(n-butylsulfonyl)piperazin-1-yl]-8-nitro-6-(trifluoromethyl)-4H-1,3-benzothiazin-4-one **3d** (11626093) Yield 64%. Mp. 215-9 °C (H_2O/EtOH). Mass (EI),

m/z (*I*$_{relat}$.(%)): 479.4839 [M]$^+$ (89). $C_{17}H_{19}F_3N_4O_5S_2$. ^{1}H NMR (DMSO-d$_6$): 0.85 (3H, t, *J* = 7.2 Hz, CH_3), 1.23 (2H, m, CH_2), 1.66 (2H, m, CH_2), 2.64 (2H, t, *J* = 7.2 Hz, CH_2), 3.21 (4H, br s, $N(CH_2)_2$), 4.06 (4H, br s, $N(CH_2)_2$), 8.75 (1H, s, CH), 8.84 (1H, s, CH) ppm.

2-[4-(Cyclohexylsulfonyl)piperazin-1-yl]-8-nitro-6-(trifluoromethyl)-4H-1,3-benzothiazin-4-one **3e** (11326127) Yield 47%. Mp. 283-5 ºC (EtOH). Mass (EI), *m/z* (*I*$_{relat}$.(%)): 463.4415 [M]$^+$ (72). $C_{19}H_{21}F_3N_4O_5S_2$. ^{1}H NMR (DMSO-d$_6$): 1.52 (6H, m, $(CH_2)_3$), 2.42 (4H, m, 2CH_2), 2.64 (2H, t, *J* = 7.2 Hz, CH_2), 3.21 (4H, br s, $N(CH_2)_2$), 4.06 (4H, br s, $N(CH_2)_2$), 8.75 (1H, s, CH), 8.84 (1H, s, CH) ppm.

2-{4-[(4-butylphenyl)sulfonyl]piperazin-1-yl}-8-nitro-6-(trifluoromethyl)-4H-1,3-benzothiazin-4-one **3f** (11326124) Yield 37%. Mp. 226-9 ºC (EtOH). Mass (EI), *m/z* (*I*$_{relat}$.(%)): 555.5799 [M]$^+$ (69). $C_{23}H_{23}F_3N_4O_5S_2$. ^{1}H NMR (DMSO-d$_6$): 0.88 (3H, t, *J* = 7.2 Hz, CH_3), 1.23 (2H, m, CH_2), 1.66 (2H, m, CH_2), 2.66 (2H, m, CH_2), 3.21 (4H, br s, $N(CH_2)_2$), 4.06 (4H, br s, $N(CH_2)_2$), 7.03 (2H, d, *J* = 8.7 Hz, 2CH), 7.66 (2H, d, *J* = 8.7 Hz, 2CH), 8.77 (1H, s, CH), 8.86 (1H, s, CH) ppm.

2-{4-[(4-trifluoromethylphenyl)sulfonyl]piperazin-1-yl}-8-nitro-6-(trifluoromethyl)-4H-1,3-benzothiazin-4-one **3g** (11326123) Yield 46%. Mp. 222-5 ºC (EtOH). Mass (EI), *m/z* (*I*$_{relat}$.(%)): 567.4715 [M]$^+$ (50). $C_{20}H_{14}F_6N_4O_5S_2$. ^{1}H NMR (DMSO-d$_6$): 3.22 (4H, br s, $N(CH_2)_2$), 4.05 (4H, br s, $N(CH_2)_2$), 7.82 (2H, d, *J* = 6.3 Hz, 2CH), 8.04 (2H, d, *J* = 6.3 Hz, 2CH), 8.72 (1H, s, CH), 8.88 (1H, s, CH) ppm.

2-{4-[(4-trifluoromethoxyphenyl)sulfonyl]piperazin-1-yl}-8-nitro-6-(trifluoromethyl)-4H-1,3-benzothiazin-4-one **3h** (11326120) Yield 52%. Mp. 207-9 ºC (EtOH). Mass (EI), *m/z* (*I*$_{relat}$.(%)): 583.4709 [M]$^+$ (36). $C_{20}H_{14}F_6N_4O_7S_2$. ^{1}H NMR (DMSO-d$_6$): 3.20 (4H, br s, $N(CH_2)_2$), 4.01 (4H, br s, $N(CH_2)_2$), 7.62 (2H, d, *J* = 6.3 Hz, 2CH), 7.90 (2H, d, *J* = 6.3 Hz, 2CH), 8.71 (1H, s, CH), 8.83 (1H, s, CH) ppm.

2-{4-[(4-acetylphenyl)sulfonyl]piperazin-1-yl}-8-nitro-6-(trifluoromethyl)-4H-1,3-benzothiazin-4-one **3i** (11326119) Yield 64%. Mp. 280-2 ºC (CH_3CN). Mass (EI), *m/z* (*I*$_{relat}$.(%)): 541.5102 [M]$^+$ (59). $C_{21}H_{17}F_3N_4O_6S_2$. ^{1}H NMR (DMSO-d$_6$): 2.48 (3H, s, CH_3), 3.26 (4H, br s, $N(CH_2)_2$), 4.03 (4H, br s, $N(CH_2)_2$), 7.97 (4H, m, 4CH), 8.71 (1H, s, CH), 8.83 (1H, s, CH) ppm.

2-(4-{[4-(1,1-dimethylpropyl)phenyl]sulfonyl}piperazin-1-yl)-8-nitro-6-(trifluoromethyl)-4H-1,3-benzothiazin-4-one **3j** (11326125) Yield 37%. Mp. 231-3 ºC (EtOH). Mass (EI), *m/z* (*I*$_{relat}$.(%)): 569.6065 [M]$^+$ (33). $C_{24}H_{25}F_3N_4O_6S_2$. ^{1}H NMR

(DMSO-d$_6$): 0.76 (3H, t, J = 7.2 Hz, CH_3), 1.31 (6H, s, 2CH_3), 1.69 (2H, m, CH_2), 3.23 (4H, br s, $N(CH_2)_2$), 4.06 (4H, br s, $N(CH_2)_2$), 7.33 (2H, d, J = 8.7 Hz, 2CH), 7.47 (2H, d, J = 8.7 Hz, 2CH), 8.73 (1H, s, CH), 8.87 (1H, s, CH) ppm.

2-{4-[(3,5-dimethylisoxazol-4-yl)sulfonyl]piperazin-1-yl}-8-nitro-6-(trifluoromethyl)-4H-1,3-benzothiazin-4-one **3k** (11326056) Yield 79%. Mp. 265-7 °C (EtOH/DMF). Mass (EI), *m/z* (I_{relat}.(%)): 518.4813 $[M]^+$ (31). $C_{18}H_{16}F_3N_5O_6S_2$. ^1H NMR (DMSO-d$_6$): 2.31 (3H, s, CH_3), 2.64 (3H, s, CH_3), 3.26 (4H, br s, $N(CH_2)_2$), 4.05 (4H, br s, $N(CH_2)_2$), 8.77 (1H, s, CH), 8.85 (1H, s, CH) ppm.

2-{4-[(1-ethyl-3-methyl-1H-pyrazol-4-yl)sulfonyl]piperazin-1-yl}-8-nitro-6-(trifluoromethyl)-4H-1,3-benzothiazin-4-one **3l** (11326058) Yield 73%. Mp. 218-20 °C (EtOH). Mass (EI), *m/z* (I_{relat}.(%)): 531.5237 $[M]^+$ (44). $C_{19}H_{19}F_3N_6O_5S_2$. ^1H NMR (DMSO-d$_6$): 1.31 (3H, t, J = 5.9 Hz, CH_3), 2.28 (3H, s, CH_3), 3.18 (4H, br s, $N(CH_2)_2$), 4.06 (4H, br s, $N(CH_2)_2$), 4.09 (2H, q, J = 6.6 Hz, CH_3), 8.27 (1H, s, CH), 8.78 (1H, s, CH), 8.86 (1H, s, CH) ppm.

8-nitro-2-[4-(2-thienylsulfonyl)piperazin-1-yl]-6-(trifluoromethyl)-4H-1,3-benzothiazin-4-one **3m** (11326059) Yield 80%. Mp. 210-4 °C (EtOH). Mass (EI), *m/z* (I_{relat}.(%)): 505.5042 $[M]^+$ (67). $C_{17}H_{13}F_3N_4O_5S_3$. ^1H NMR (DMSO-d$_6$): 3.20 (4H, br s, $N(CH_2)_2$), 4.05 (4H, br s, $N(CH_2)_2$), 7.25 (1H, br s, CH), 7.68 (1H, br s, CH), 8.03 (1H, br s, CH), 8.73 (1H, s, CH), 8.83 (1H, s, CH) ppm.

2-{4-[(1,3-dimethyl-1H-pyrazol-4-yl)sulfonyl]piperazin-1-yl}-8-nitro-6-(trifluoromethyl)-4H-1,3-benzothiazin-4-one **3n** (11326061) Yield 64%. Mp. 200-3 °C (EtOH). Mass (EI), *m/z* (I_{relat}.(%)): 517.4966 $[M]^+$ (74). $C_{18}H_{17}F_3N_6O_5S_2$. ^1H NMR (DMSO-d$_6$): 2.41 (3H, s, CH_3), 3.17 (4H, br s, $N(CH_2)_2$), 3.72 (3H, s, NCH_3), 4.03 (4H, br s, $N(CH_2)_2$), 7.63 (s, 1H, CH), 8.75 (1H, s, CH), 8.86 (1H, s, CH)ppm.

2-{4-[(5-chloro-2-thienyl)sulfonyl]piperazin-1-yl}-8-nitro-6-(trifluoromethyl)-4H-1,3-benzothia-zin-4-one **3o** (11326121) Yield 39%. Mp. 210-3 °C (EtOH/DMF). Mass (EI), *m/z* (I_{relat}.(%)): 539.9492 $[M]^+$ (53). $C_{17}H_{12}F_3ClN_4O_5S_3$. ^1H NMR (DMSO-d$_6$): 3.24 (4H, br s, $N(CH_2)_2$), 4.06 (4H, br s, $N(CH_2)_2$), 7.39 (1H, d, J = 5.1 Hz, CH), 7.58 (1H, d, J = 5.1 Hz, CH),, 8.78 (1H, s, CH), 8.85 (1H, s, CH) ppm.

2-{4-[(4,5-dichloro-2-thienyl)sulfonyl]piperazin-1-yl}-8-nitro-6-(trifluoromethyl)-4H-1,3-benzothia-zin-4-one **3p** (11326122) Yield 46%. Mp. 265-7 °C (EtOH/DMF). Mass (EI), *m/z* (I_{relat}.(%)): 574.3942 $[M]^+$ (58). $C_{17}H_{11}F_3Cl_2N_4O_5S_3$. ^1H NMR (DMSO-

d_6): 3.26 (4H, br s, N(CH_2)$_2$), 4.07 (4H, br s, N(CH_2)$_2$), 7.83 (1H, s, CH), 8.77 (1H, s, CH), 8.86 (1H, s, CH) ppm.

2-{4-[(3-methylisoxazol-4-yl)sulfonyl]piperazin-1-yl}-8-nitro-6-(trifluoromethyl)-4H-1,3-benzothiazin-4-one **3q** (11326126) Yield 86%. Mp. 281-3 ºC (EtOH/DMF). Mass (EI), *m/z* (I_{relat}.(%)): 504.4542 $[M]^+$ (61). $C_{17}H_{14}F_3N_5O_6S_2$. ^{1}H NMR (DMSO-d_6): 2.33 (3H, s, CH_3), 3.26 (4H, br s, N(CH_2)$_2$), 4.04 (4H, br s, N(CH_2)$_2$), 7.69 (1H, s, CH), 8.75 (1H, s, CH), 8.86 (1H, s, CH) ppm.

Methyl 3-({4-[8-nitro-4-oxo-6-(trifluoromethyl)-4H-1,3-benzothiazin-2-yl]piperazin-1-yl}sulfo-nyl)thiophene-2-carboxylate **3r** (11326128) Yield 74%. Mp. 127-9 ºC (EtOH/DMF). Mass (EI), *m/z* (I_{relat}.(%)): 563.5412 $[M]^+$ (68). $C_{19}H_{15}F_3N_4O_7S_3$. ^{1}H NMR (DMSO-d_6): 3.20 (4H, br s, N(CH_2)$_2$), 3.83 (3H, s, OCH_3), 4.07 (4H, br s, N(CH_2)$_2$), 6.81 (1H, d, J = 5.3 Hz, CH), 7.67 (1H, d, J = 5.3 Hz, CH), 8.70 (1H, s, CH), 8.84 (1H, s, CH) ppm.

2-[4-(isoquinolin-5-ylsulfonyl)piperazin-1-yl]-8-nitro-6-(trifluoromethyl)-4H-1,3-benzothiazin-4-one **3s** (11626094) Yield 85%. Mp. 243-5 ºC (EtOH/DMF). Mass (EI), *m/z* (I_{relat}.(%)): 550.5265 $[M]^+$ (36). $C_{22}H_{16}F_3N_5O_5S_2$. ^{1}H NMR (DMSO-d_6): 3.24 (4H, br s, N(CH_2)$_2$), 4.06 (4H, br s, N(CH_2)$_2$), 7.80 (1H, t, J = 7.9 Hz, CH), 7.99 (1H, d, J = 5.6 Hz, CH), 8.38 (2H, m, 2CH), 8.73 (1H, s, CH), 8.85 (1H, s, CH), 8.93 (1H, d, J = 5.6 Hz, CH), 9.57 (1H, s, CH) ppm.

Table S1 : IC50 values measured for Ty38c, PBTZ169 and sPBTZ169 (11326127) against wild-type and BTZ-resistant *M. tuberculosis* C387S DprE1

compound	structure	IC_{50} (μM)		References
		WT	C387S	
PBTZ169	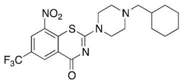	0.3	3.6	This study
Ty38c	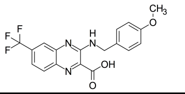	0.2	0.2	(1)
sPBTZ169	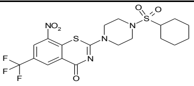	1.1	12	This study

Table S2. Data collection and refinement statistics

	DprE1-sPBTZ169
Beamline	SLS X06DA (PXIII)
Wavelength (Å)	1
Resolution range (Å)	49.15- 2.40 (2.48-2.40)
Space group	P 1 2_1 1
Unit cell (Å, °)	78.164 84.024 81.666 90 103.983 90
Total reflections	79124 (7781)
Unique reflections	39831 (3914)
Multiplicity	2.0 (2.0)
Completeness (%)	98.81 (98.19)
Mean I/sigma(I)	16.42 (2.56)
R-merge	0.03362 (0.3709)
R-meas	0.04754 (0.5245)
CC1/2	0.999 (0.85)
CC*	1 (0.959)
R-work	0.1978
R-free	0.2248
Number of non-hydrogen atoms	6512
Ramachandran favored (%)	97.58
Ramachandran allowed (%)	2.42
Ramachandran outliers (%)	0.00
Rotamer outliers (%)	1.92
Clashscore	4.47
Average B-factor	60.34

Statistics for the highest-resolution shell are shown in parentheses.

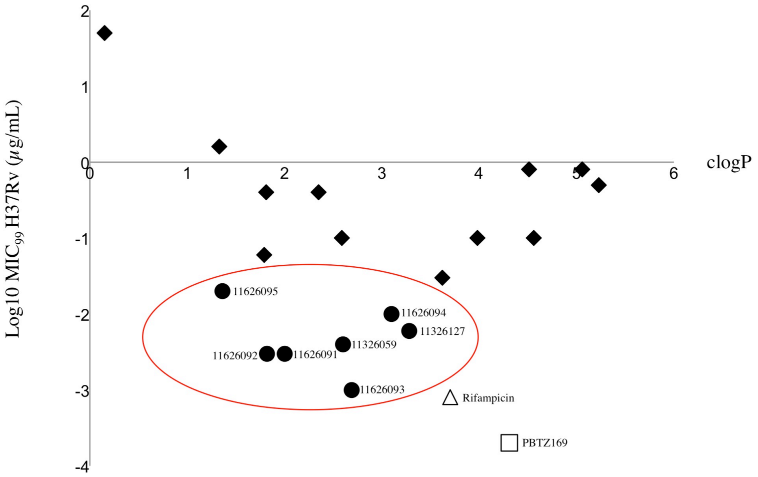

Fig S1: **Antitubercular activity versus hydrophobicity.** Distribution and activity of 2-sulphonylpiperazin 8-nitro 6-trifluoromethyl 1,3-benzothiazin-4-ones derivatives as a function of clogP. Surrounded in red, the 7 chosen compounds with a good activity/logP profile. Lozenges indicate other compounds not discussed in the text.

References

1. Foo CS, Lechartier B, Kolly GS, Boy-Rottger S, Neres J, Rybniker J, Lupien A, Sala C, Piton J, Cole ST. 2016. Characterization of DprE1-Mediated Benzothiazinone Resistance in Mycobacterium tuberculosis. Antimicrob Agents Chemother 60:6451-6459.
